# Physical activity, chronic disease, depression, and health-related quality of life among Chinese older adults: a two-wave longitudinal panel study before and during COVID-19

**DOI:** 10.3389/fpubh.2026.1847304

**Published:** 2026-05-13

**Authors:** Chaoqi Li, Zixiang Zhou, Kyungsik Kim

**Affiliations:** 1 Medical and Health College, Xuchang Vocational Technical College, Xuchang, China; 2 School of Physical Education, Hunan University of Science and Technology, Xiangtan, China; 3 Department of Sport & Leisure Studies, Hoseo University, Asan-si, Republic of Korea

**Keywords:** chronic diseases, COVID-19, depression, health-related quality of life, older adults, physical activity

## Abstract

**Background:**

Population aging and the COVID-19 pandemic have intensified the physical and mental health challenges of older adults in China, highlighting the need for sustainable health behavior strategies. While physical activity (PA) is an important modifiable factor, the impact of different PA intensity levels and the mechanisms of this influence on physical and mental health remain unclear. We examined the associations between PA, chronic disease, depression, and health-related quality of life (HRQoL) before and during the pandemic.

**Methods:**

Data were obtained from the China Health and Retirement Longitudinal Study (CHARLS) conducted in 2018 and 2020, including 21,425 adults aged ≥ 60 years. Comparative analysis and path analysis were used to assess the associations among PA, chronic disease (i.e., hypertension, dyslipidemia, and diabetes), depression, and HRQoL, as well as the direct and indirect associations of PA on HRQoL. Participants were older adults who engaged in PA at least twice per week.

**Results:**

Older adults in China experienced a decline in health status during the pandemic, with higher prevalence of chronic disease, more severe depression symptoms, and reduced HRQoL. In both periods, moderate- and vigorous-intensity PA related to lower chronic disease prevalence, whereas moderate- and low-intensity PA related to less severe depression symptoms. Path analysis indicates that PA related to HRQoL through both physical and mental health pathways, with depression symptoms acting as a key mediator. The indirect mental health benefits of low-intensity PA were more pronounced during the pandemic.

**Conclusion:**

Regular moderate- and low-intensity PA relates to favorable physical and mental health outcomes in older adults during public health crises. Integrating goal-oriented and intensity-matched PA programs into community-based care and emergency response systems might support physical and mental resilience in future emergencies and improve health sustainability.

## 1 Introduction

Population aging is one of the most profound demographic trends of the 21st century, and China is experiencing this transition at an unprecedented pace. By the end of 2024, China’s older population (i.e., ≥ 60 years old) had reached 310 million, accounting for 22% of the total population (1). This rapid shift poses challenges to health system sustainability, the ability to maintain population health and provide essential services through core functions, even in the face of demographic changes, resource constraints, and public health challenges (2). In the context of an aging society, which generates continual growth in the health management and care needs of older adults, health system sustainability represents an important way to measure the long-term carrying capacity of health intervention.

The changing demographics of China have further highlighted a series of specific health challenges faced by older adults. According to statistics from the National Health Commission of China (3), nearly 180 million older adults (75%) suffer from one or more chronic diseases. Mental health issues are also prevalent among older adults, with depression having a significant impact on mental well-being and daily functioning (3). This dual impairment has led to a general decline in health-related quality of life (HRQoL) (4). In response to these challenges, China has launched initiatives such as “Healthy China 2030” to strengthen health promotion and enhance community-based primary healthcare services (5). This policy is consistent with the United Nations Sustainable Development Goal 3 (SDG 3), which emphasizes the importance of ensuring healthy lifestyles for all ages (6). However, China’s current policies have several shortcomings. In addition to lack of access to medical resources, significant lack of attention to sustainable health behaviors is evident (7, 8). In this context, addressing the health challenges of older adults requires improving health sustainability, at the system level and in the individual. While health system sustainability involves the capacity of healthcare services and resources (2), individual health sustainability in aging adults involves the ability to maintain healthy behaviors, adapt to functional decline, and cope with external shocks (9). Identifying effective and sustainable health behaviors that address both physical and mental health challenges is essential. Among these behaviors, physical activity (PA) has received particular attention due to its broad health benefits and feasibility for older adults (10).

PA is widely regarded as one of the most cost-effective strategies for managing chronic diseases and promoting long-term health (11–13). Previous findings show that regular PA can improve cardiovascular function and metabolic health and reduce the risk of multiple chronic diseases in older adults (14, 15). The benefits of PA are not limited to physical health; it also plays an important role in promoting mental health. Findings suggest that regular PA can alleviate depression symptoms in older adults and, through this mediation pathway, improve cognitive function and HRQoL (16, 17). Meanwhile, PA can also have a positive impact on overall mental health in older adults by promoting social participation and providing positive and pleasurable experiences (18, 19).

An integrated framework combining Activity Theory, Successful Aging Theory, and Self-Determination Theory (SDT) can shed light on the relationship between PA and health outcomes in later life. Activity Theory posits that ongoing engagement in physical and social activities contributes to greater life satisfaction and psychological well-being in older adults (20, 21). Successful Aging Theory further posits that maintaining physical function, preventing chronic disease, and sustaining psychological well-being are key components of healthy aging (22). This theoretical framework suggests that PA operates through interconnected pathways involving physical health and psychological well-being, ultimately improving HRQoL in later life (14, 23). Complementing these perspectives, SDT focuses on the motivational mechanisms underlying sustained engagement in PA, suggesting that intrinsic motivation, autonomy, and perceived competence play essential roles in maintaining long-term health behaviors (24, 25).

These theories provide a comprehensive framework in which PA functions as a central behavioral pathway linking motivation (SDT), active engagement (Activity Theory), and multidimensional health outcomes (Successful Aging Theory). PA might influence health-related quality of life both directly and indirectly through its effects on chronic disease and depression. This integrated framework not only explains how PA contributes to physical and mental health but also provides a theoretical basis for understanding how PA sustains individual health, particularly under stressful conditions such as the COVID-19 pandemic.

The COVID-19 pandemic posed a critical stress test for health sustainability, exposing the limits of health systems and compromising the ability of individuals to maintain healthy behaviors amid rapid environmental changes (26, 27). Governments worldwide implemented lockdowns and social restrictions, with China adopting particularly strict measures, which negatively affected the physical and mental health of older adults (28). During this period, PA levels declined, while sedentary behavior, loneliness, and depression symptoms increased and social participation and HRQoL decreased (29–31). These changes highlight the vulnerability of older adult health behaviors to external shocks and underscore the importance of examining how sustainable health behaviors influence physical and mental health. Such disruptions to regular PA can limit opportunities for social and physical engagement and interfere with the multidimensional processes of maintaining health and well-being. In this context, maintaining healthy behaviors and adaptive capacity among older adults is critical (32).

Although previous findings show the relationship between PA and health in older adults, most evidence is tied to cross-sectional designs and limited to specific regions or small samples (16, 33, 34). Few scholars have used nationally representative longitudinal data to compare changes before and during the pandemic. In addition, many scholars have not distinguished between different PA intensities, limiting the understanding of their differential effects. Furthermore, most previous studies have addressed the simple associations between PA and HRQoL, with limited attention to underlying pathways involving chronic disease and depression. As a result, the mechanisms linking PA to HRQoL remain unclear, particularly in the context of the pandemic.

To fill this gap, we used nationally representative longitudinal data collected by the China Health and Retirement Longitudinal Study (CHARLS) before the COVID-19 outbreak (2018) and during the pandemic (2020) to examine the relationship between different intensities of PA, chronic disease, depression, and HRQoL among older adults in China. Furthermore, we constructed a model to distinguish between direct and indirect pathways between PA of different intensities and these health outcomes, elucidating the protective role of PA in individual health sustainability before and after the pandemic. The findings of this study should provide (a) empirical evidence for an integrated theoretical framework combining Activity Theory, Successful Aging Theory, and SDT in the special context of public health crises and (b) a scientific basis for targeted policies that promote healthy aging and sustain long-term development of China’s health system.

Accordingly, we proposed the following hypotheses:

*H*1. Chronic diseases will differ according to PA level before and during COVID-19.

*H*2. Depression will differ according to PA level before and during COVID-19.

*H*3. HRQoL will differ according to PA level before and during COVID-19.

*H*4. Depression before and during COVID-19 will mediate the relationship between PA level and HRQoL.

## 2 Materials and methods

### 2.1 Participants

Our sample consisted of Chinese adults aged 60 and above. We obtained authorization to use the 2018 and 2020 CHARLS data after submitting a research proposal to Peking University’s National School of Development. As a nationally representative longitudinal panel survey administered by Peking University, CHARLS targets Chinese residents aged 45 and older, with a multistage PPS random sampling design (implicitly stratified by region, urban–rural status, and per capita GDP) covering 28 provinces, 150 counties/districts, and 450 villages/communities (35).

To examine the impact of COVID-19 on older adults, we defined 2018 as the period “before COVID-19” and 2020 as the period “during COVID-19.” Because CHARLS has a longitudinal panel design, the same cohort of respondents and their spouses participated in both surveys, enabling within-subject comparisons over time. Participants were included if they met the following criteria: (a) aged ≥60 years and (b) had complete data on PA, depression, chronic diseases, HRQoL. The initial CHARLS samples included 19,816 respondents in 2018 and 19,395 in 2020. After excluding those younger than 60 years old and those with incomplete baseline information, the final analytic sample included 10,771 participants in 2018 and 10,654 in 2020. Although minor differences in sample size occurred due to attrition (e.g., death, no follow-up) and new eligibility (e.g., participants reaching age 60 by 2020, recontacted individuals), the analytic dataset remained an unbalanced longitudinal panel, rather than two independent cross-sectional samples. Attrition resulted in a loss of 1,242 participants, while 1,125 new individuals were added to the panel.

**Figure 1:**
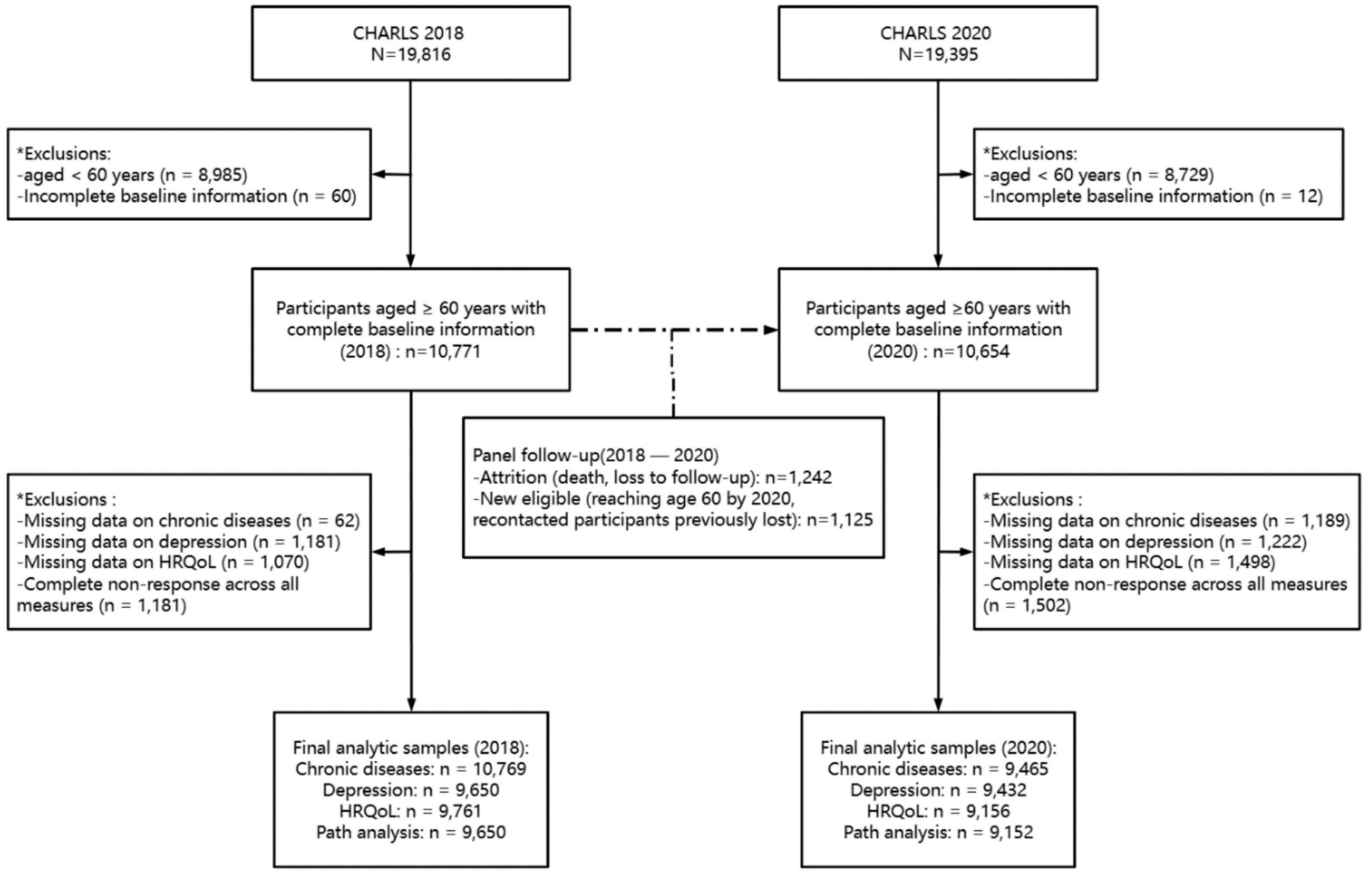
Flow diagram of participant selection and follow-up (CHARLS 2018–2020 longitudinal panel design).

Due to item non-response, the number of participants included in specific analyses varied. Chronic disease analysis included 10,769 participants in 2018 and 9,465 in 2020; depression analysis included 9,650 participants in 2018 and 9,432 in 2020; HRQoL analysis included 9,761 participants in 2018 and 9,156 in 2020; and path analysis included 9,650 participants in 2018 and 9,152 in 2020 (see Figure 1).

### 2.2 Measurements

#### 2.2.1 Physical activity

PA among older adults in the CHARLS dataset was measured using a localized short form of the International Physical Activity Questionnaire (IPAQ), an internationally validated instrument for assessing PA during the preceding 7 days (36). The questionnaire classifies activities into three intensity levels: vigorous-, moderate-, and low-intensity. Vigorous-intensity PA refers to activities that cause noticeable shortness of breath, such as lifting heavy objects, digging, tilling, aerobic exercise, fast cycling, or riding a freight bike. Moderate-intensity PA includes activities that cause breathing to be slightly faster than usual (e.g., lifting light objects, cycling at a normal pace, mopping the floor, practicing Tai Chi, or brisk walking). Low-intensity PA includes walking or strolling, moving from one place to another at work or at home, and other walking activities undertaken for leisure, exercise, training, or recreation. Participants were asked to report how many days in the previous week they had engaged in at least 10 min of this type of activity and the average duration per day. Respondents who had engaged in any form of PA at least twice a week were categorized as PA participants, while those who had exercised less than twice a week were categorized as non-participants. These criteria are consistent with the recommendations of the Korean Ministry of Culture, Sports, and Tourism, which considers at least two physical activities per week to be an important criterion in its annual physical participation survey (37, 38). This recommendation indicates that engaging in PA at least twice a week is necessary to obtain the benefits of exercise.

#### 2.2.2 Chronic diseases

Hypertension, dyslipidemia, and diabetes were selected as target chronic diseases because they are highly prevalent among Chinese older adults, strongly associated with physical activity and aging, and commonly examined in large-scale population-based longitudinal studies (35, 39–41). Their inclusion in this study provides a representative and theoretically grounded measure of chronic disease burden in older populations. The prevalence of chronic diseases was determined through self-reporting. Participants were asked whether they had ever been diagnosed with any of these three diseases, responding with “yes” or “no.” To ensure that only formally diagnosed cases were included in the analysis, CHARLS compared the results of the inquiry with medical history data collected in the baseline health survey to determine chronic disease prevalence (35, 42). This approach reduced false positives and ensured that only medically confirmed participants were counted as having chronic diseases.

#### 2.2.3 Depression

We used the Center for Epidemiological Research Depression Scale-10 (CESD-10) to assess depression symptoms. The CESD-10 contains 10 questions covering multiple dimensions (e.g., mood and behavior). A four-point scale was used to assess the frequency of each symptom among the participants over the previous week: 0 = “rarely or never (< 1 day),” 1 = “sometimes (1-2 days),” 2 = “occasionally (3–4 days),” 3 = “most or all of the time (5–7 days).” The two positive emotion questions, “I feel happy” and “I am hopeful for the future,” were reverse scored. All scores were added together to obtain a total score, ranging from 0 to 30 points. Higher scores meant more severe depression symptoms (43).

#### 2.2.4 Health-related quality of life

We used the EuroQol five-dimensional three-level questionnaire (EQ-5D-3L) to assess HRQoL among older adults in China. The EQ-5D-3L evaluates health across five dimensions—mobility, self-care, usual activities, pain/discomfort, and anxiety—each with three levels: no problems, some problems, and severe problems (scored as 1, 2, or 3, respectively) (44). In this study, mobility was assessed using questions about difficulty getting into or out of bed and using the toilet. Self-care covered difficulties in dressing, bathing/showering, eating, and controlling urination/defecation. Usual activities included the ability to work and do housework. Pain/discomfort was evaluated by the frequency and severity of body pain. Anxiety was measured by feelings of fear experienced during the past week.

Health utility values (HUVs) were then calculated using the HRQoL composite scoring system developed by Liu et al. (45) for the Chinese population. This system converts responses across the three levels of each EQ-5D-3L dimension into a health utility score, representing overall HRQoL among older adults. Scores ranged from 0 to 1, with higher values (closer to 1) indicating better HRQoL. The calculation formula is the following:


$$U=1\sum_{i=1}^{5}(w_{i}\times (\text{Leve}l_{i}-1))-N3$$

*U* represents the Health Utility Value; *w* represents the specific weighting coefficients for each of the five dimensions (45); *Level* represents the reported level (1, 2, or 3) for each dimension; and *N3* represents an additional penalty applied when any dimension has a level of 3, indicating a severe problem.

### 2.3 Statistical analyses

All statistical analyses were performed using IBM SPSS Statistics 26.0. Chi-square tests were used to examine differences in chronic disease prevalence across PA groups. Two-way ANOVAs were used to assess the main and interaction effects of PA intensity on depression and HRQoL. In addition, path analysis was used to examine depression as a mediating variable in the relationship between PA and HRQoL. These methods allowed us to investigate both the direct effects of PA on HRQoL and the indirect effects mediated through depression.

Cramer’s V was applied to determine the effect size (ES) in the cross-tabulation analysis. Values closer to 0 indicate weaker associations, whereas values approaching 1 reflect stronger relationships. Effect sizes for the two-way ANOVA were assessed using partial eta squared as provided in the SPSS output; values of 0.01, 0.06, and 0.14 were interpreted as small, medium, and large effects, respectively. Statistical significance was set at *p* < 0.05 for all tests.

### 2.4 Ethical considerations

This study adhered to Institutional Review Board (IRB) guidelines. Ethical approval for all waves of the CHARLS survey was obtained from the Biomedical Ethics Committee of Peking University (Approval Number: IRB00001052-11015). During the field surveys, all participants who provided consent were asked to sign two copies of the informed consent form—one retained by the participant and the other stored as a PDF file at the CHARLS office. To ensure full alignment with established research ethics, this study also received additional ethical clearance from the Institutional Review Board of Hunan University of Science and Technology (Approval Number: HT2025051) on 8 March 2025.

## 3 Results

### 3.1 Differences in chronic diseases by PA before and during COVID-19

#### 3.1.1 Differences in hypertension

As shown in Table 1, the prevalence of hypertension was higher during COVID-19 (49.2%) than before COVID-19 (47.2%). For vigorous-intensity PA, non-participants had a higher prevalence of hypertension than participants in both years (before COVID-19: 38.1% vs. 9.2%; *p* < 0.001, ES = 0.098; during COVID-19: 36.6% vs. 12.6%; *p* < 0.001, ES = 0.079). A similar pattern emerged for moderate-intensity PA, with consistently lower prevalence among participants than non-participants (*p* < 0.001, ES = 0.048 in 2018; *p* < 0.001, ES = 0.066 in 2020). In the low-intensity PA group, participants had a higher prevalence of hypertension than non-participants before COVID-19 (36.6% vs. 10.6%; *p* < 0.001, ES = 0.037), but this difference was no longer significant during COVID-19. This shift suggests that low-intensity PA might not provide sufficient support for health sustainability in terms of hypertension prevention under pandemic conditions, highlighting the need for intensity-specific recommendations in public health interventions targeting older adults during crises. From the perspective of Successful Aging Theory, maintaining physiological resilience requires sufficient intensity of health behaviors, potentially explaining why low-intensity PA showed limited effectiveness under stressful conditions such as COVID-19.

**Table 1:** Differences in chronic diseases.

Disease	PA		Before COVID-19 (2018)		During COVID-19 (2020)		*p*-value
			Experience	Inexperience	Experience	Inexperience	
Hypertension	Vigorous PA	Par	987 (9.2%)	1,576 (14.6%)	1,194 (12.6%)	1,579 (16.7%)	103.018***
		Non-par	4,101 (38.1%)	4,105 (38.1%)	3,465 (36.6%)	3,227 (34.1%)	59.649***
	Moderate PA	Par	1,920 (17.8%)	2,411 (22.4%)	2,079 (22.0%)	2,460 (26.0%)	24.701***
		Non-par	3,168 (29.4%)	3,270 (30.4%)	2,580 (27.2%)	2,346 (24.8%)	40.823***
	Low PA	Par	3,945 (36.6%)	4,577 (42.5%)	3,468 (36.6%)	3,654 (38.6%)	14.938***
		Non-par	1,143 (10.6%)	1,104 (10.3%)	1,191 (12.6%)	1,152 (12.2%)	3.225
Dyslipidemia	Vigorous PA	Par	475 (4.4%)	2,088 (19.4%)	652 (6.9%)	2,121 (22.4%)	65.410***
		Non-par	2,167 (20.1%)	6,039 (56.1%)	2,251 (23.8%)	4,441 (46.9%)	94.517***
	Moderate PA	Par	1,056 (9.8%)	3,275 (30.4%)	1,359 (14.4%)	3,180 (33.6%)	0.089
		Non-par	1,586 (14.7%)	4,852 (45.1%)	1,544 (16.3%)	3,382 (35.7%)	2.188
	Low PA	Par	2,131 (19.8%)	6,391 (59.3%)	2,231 (23.6%)	4,891 (51.6%)	4.925*
		Non-par	511 (4.7%)	1,736 (16.1%)	672 (7.1%)	1,671 (17.7%)	5.797*
Diabetes	Vigorous PA	Par	280 (2.6%)	2,283 (21.2%)	407 (4.3%)	2,366 (25.0%)	51.249***
		Non-par	1,376 (12.8%)	6,830 (63.4%)	1,329 (14.0%)	5,363 (56.7%)	35.156***
	Moderate PA	Par	628 (5.8%)	3,703 (34.4%)	787 (8.3%)	3,752 (39.6%)	4.285*
		Non-par	1,028 (9.5%)	5,410 (50.2%)	949 (10.0%)	3,977 (42.0%)	5.854*
	Low PA	Par	1,304 (12.1%)	7,218 (67.0%)	1,325 (14.0%)	5,797 (61.2%)	0.181
		Non-par	352 (3.3%)	1,895 (17.6%)	411 (4.3%)	1,932 (20.4%)	1.329

*p < 0.05, **p < 0.01, ***p < 0.001; Par, Participants; Non-par, Non-participants. For every PA intensity group, the upper entry in the p-value column denotes the statistical significance of differences between participants and non-participants in 2018, whereas the lower entry represents the significance of such differences in 2020.

#### 3.1.2 Differences in dyslipidemia

As shown in Table 1, the prevalence of dyslipidemia increased from 24.5% before COVID-19 to 30.7% during COVID-19. For vigorous-intensity PA, non-participants consistently showed a higher prevalence of dyslipidemia than participants in both years (before COVID-19: 20.1% vs. 4.4%; *p* < 0.001, ES = 0.078; during COVID-19: 23.8% vs. 6.9%; *p* < 0.001, ES = 0.100;), demonstrating that vigorous PA maintained a robust and sustainable protective effect, thereby supporting health sustainability by mitigating dyslipidemia risk even as overall prevalence increased. For moderate-intensity PA, prevalence was also lower among participants than non-participants in both years, though the differences were smaller (before COVID-19: 9.8% vs. 14.7%; during COVID-19: 14.4% vs. 16.3%), indicating that moderate PA retained a modest but clinically relevant role in promoting health sustainability given the pandemic-related rise in metabolic risk. In contrast, for low-intensity PA, participants had a higher prevalence than non-participants in both years (*p* < 0.05, ES = 0.021 in 2018; *p* < 0.05, ES = 0.025 in 2020), suggesting that low-intensity PA might not be a viable strategy for supporting metabolic health sustainability in older adults, even in non-crisis periods.

#### 3.1.3 Differences in diabetes

As shown in Table 1, the prevalence of diabetes increased from 15.4% before COVID-19 to 18.3% during COVID-19. For vigorous-intensity PA, non-participants had a higher prevalence of diabetes than participants in both years (before COVID-19: 12.8% vs. 2.6%; *p* < 0.001, ES = 0.069; during COVID-19: 14.0% vs. 4.3%; *p* < 0.001, ES = 0.061), demonstrating that vigorous PA maintained a sustainable protective effect against diabetes, thereby contributing to health sustainability even amid pandemic-related disruptions in daily life. For moderate-intensity PA, non-participants had a higher prevalence of diabetes than participants in both years (before COVID-19: 9.5% vs. 5.8%; *p* < 0.05, ES = 0.020; during COVID-19: 10.0% vs. 8.3%; *p* < 0.05, ES = 0.025), indicating that moderate PA retained its protective role as an accessible strategy for promoting metabolic health sustainability among older adults during crises. For low-intensity PA, participants had a higher prevalence of diabetes than non-participants in both years, but the differences were not significant (*p* > 0.05), suggesting that low-intensity PA does not provide meaningful support for diabetes-related health sustainability in this population.

### 3.2 Differences in depression by PA before and during COVID-19

As shown in Table 2, across all PA intensity groups, depression scores (M) were higher during COVID-19 than before COVID-19. For vigorous-intensity PA, scores increased from M = 9.116 to M = 9.736 among participants and from M = 8.541 to M = 9.213 among non-participants. Variance analysis indicated significant main effects of PA participation (*F* = 26.438, *p* < 0.001, ES = 0.001) and year (*F* = 36.576, *p* < 0.001, ES = 0.002), with no significant interaction (*F* = 0.059, *p* = 0.809). These findings indicate that, contrary to expectations, vigorous PA participants had a higher depression score than non-participants, suggesting that vigorous-intensity PA might not have been protective for mental health. Interpreted through SDT, this pattern suggests that vigorous-intensity PA might have reduced perceived autonomy or increased psychological burden among older adults, potentially limiting its mental health benefits. For moderate-intensity PA, scores rose from M = 8.387 to M = 9.284 among participants and from M = 8.905 to M = 9.442 among non-participants, with significant main effects of PA (*F* = 12.541, *p* < 0.001, ES = 0.001) and year (*F* = 56.420, *p* < 0.001, ES = 0.003); the PA × year interaction approached significance (*F* = 3.533, *p* = 0.060). For low-intensity PA, scores increased from M = 8.444 to M = 9.080 among participants and from M = 9.678 to M = 10.239 among non-participants, with significant main effects of PA (*F* = 27.310, *p* < 0.001, ES = 0.006) and year (*F* = 109.187, *p* < 0.001, ES = 0.001), but no significant interaction (*F* = 0.105, *p* = 0.746). Notably, moderate- and low-intensity PA participants consistently reported lower depression scores than non-participants across both periods, demonstrating their critical role in promoting mental health sustainability, even under pandemic stress, in older adults who might face barriers to higher-intensity PA. This finding supports SDT: more accessible and intrinsically motivating forms of activity have greater positive effects on psychological well-being.

**Table 2:** Two-way ANOVA for differences in depression by PA before and during COVID-19.

Variables		n	Year		F value
			Before COVID-19 (2018)	During COVID-19 (2020)	
Vigorous PA	Participant	5,189	9.116 ± 6.593	9.736 ± 6.442	26.438*** 36.576*** 0.059
	Non-participant	13,893	8.541 ± 6.624	9.213 ± 6.498	
Moderate PA	Participant	8,614	8.387 ± 6.498	9.284 ± 6.530	12.541*** 56.42*** 3.533
	Non-participant	10,468	8.905 ± 6.702	9.442 ± 6.444	
Low PA	Participant	14,864	8.444 ± 6.534	9.080 ± 6.419	27.31*** 109.187*** 0.105
	Non-participant	4,218	9.678 ± 6.879	10.239 ± 6.610	

***p < 0.001. The F-values (p) for PA, year, and their interaction are presented in the first, second, and third rows of the F-value column, respectively.

### 3.3 Differences in HRQoL by PA before and during COVID-19

As shown in Table 3, HUVs across all PA intensity groups were lower during COVID-19 than before COVID-19. For vigorous-intensity PA, HUVs decreased from M = 0.766 to M = 0.698 among participants and from M = 0.710 to M = 0.662 among non-participants. Variance analysis revealed significant main effects of PA type (*F* = 152.649, *p* < 0.001, ES = 0.008) and year (*F* = 240.200, *p* < 0.001, ES = 0.013), as well as a significant PA × year interaction (*F* = 6.515, *p* = 0.011). For moderate-intensity PA, HUVs fell from M = 0.758 to M = 0.696 among participants and from M = 0.699 to M = 0.651 among non-participants, with significant effects of PA (*F* = 243.377, *p* < 0.001, ES = 0.013) and year (*F* = 275.729, *p* < 0.001, ES = 0.014) and a significant interaction (*F* = 4.353, *p* = 0.037). For low-intensity PA, HUVs declined from M = 0.746 to M = 0.687 among participants and from M = 0.635 to M = 0.628 among non-participants, with significant main effects of PA (*F* = 453.773, *p* < 0.001, ES = 0.023) and year (*F* = 68.596, *p* < 0.001, ES = 0.004) and a notably large interaction effect (*F* = 42.036, *p* < 0.001, ES = 0.002). Overall, HUVs were consistently higher among PA participants than non-participants, underscoring the positive association between PA and HRQoL. This finding aligns with the integrated theoretical framework, which posits that PA promotes health-related quality of life through both active engagement and multidimensional health maintenance. The significant PA × year interactions across all intensity groups indicate complex, dynamic changes in the influence of PA on HRQoL over time.

**Table 3:** Two-way ANOVA for differences in HRQoL by PA before and during COVID-19.

Variables		n	Year		F value
			Before COVID-19 (2018)	During COVID-19 (2020)	
Vigorous PA	Participant	5,132	0.766 ± 0.165	0.698 ± 0.218	152.649*** 240.2*** 6.515*
	Non-participant	13,785	0.710 ± 0.221	0.662 ± 0.259	
Moderate PA	Participant	8,542	0.758 ± 0.173	0.696 ± 0.223	243.377*** 275.729*** 4.353*
	Non-participant	10,375	0.699 ± 0.231	0.651 ± 0.267	
Low PA	Participant	14,762	0.746 ± 0.187	0.687 ± 0.235	453.773*** 68.596*** 42.036***
	Non-participant	4,155	0.635 ± 0.269	0.628 ± 0.280	

*p < 0.05; ***p < 0.001. The F-values (p) for PA, year, and their interaction are presented in the first, second, and third rows of the F-value column, respectively.

### 3.4 Association of PA with depression and HRQoL before and during COVID-19

Before COVID-19, the number of days engaged in PA positively related to HRQoL across all intensities, with standardized coefficients of *β* = 0.104 for vigorous-, *β* = 0.070 for moderate-, and *β* = 0.137 for low-intensity PA. Vigorous-intensity PA positively related to depression (*β* = 0.044), whereas moderate- (*β* = −0.040) and low-intensity PA (*β* = −0.078) negatively related to depression. The inverse relationship between lower-intensity PA and depression is relevant to health sustainability. Low-intensity PA is often more accessible for older adults and relates to better mental health and sustainable health outcomes. Depression negatively related to HRQoL (*β* = −0.581), and PA, together with depression, explained 39.3% of the variance in HRQoL. However, PA explained little variance in depression, suggesting that other factors might also be involved.

During COVID-19, PA continued to relate to HRQoL, although the magnitude fell across all intensities (vigorous: *β* = 0.083; moderate: *β* = 0.049; low: *β* = 0.036). The negative impact of depression on HRQoL strengthened (*β* = −0.659), increasing the explained variance in HRQoL to 45.0%. Vigorous-intensity PA remained positively related to depression (*β* = 0.035), while moderate-intensity PA showed a small and non-significant negative relationship (*β* = −0.021) and low-intensity PA maintained a significant negative relationship (*β* = −0.095).

Path model estimates indicate that before COVID-19, all PA intensities directly related to HRQoL, with low- and moderate-intensity PA also having an indirect impact via less severe depression symptoms. Low-intensity PA had the strongest positive relationship (*β* = 0.182), including direct (*β* = 0.137) and indirect through depression (*β* = 0.045). Moderate-intensity PA positively related to HRQoL (*β* = 0.093), and vigorous-intensity PA had the weakest impact (*β* = 0.078) due to a negative indirect relationship through more severe depression symptoms.

**Figure 2:**
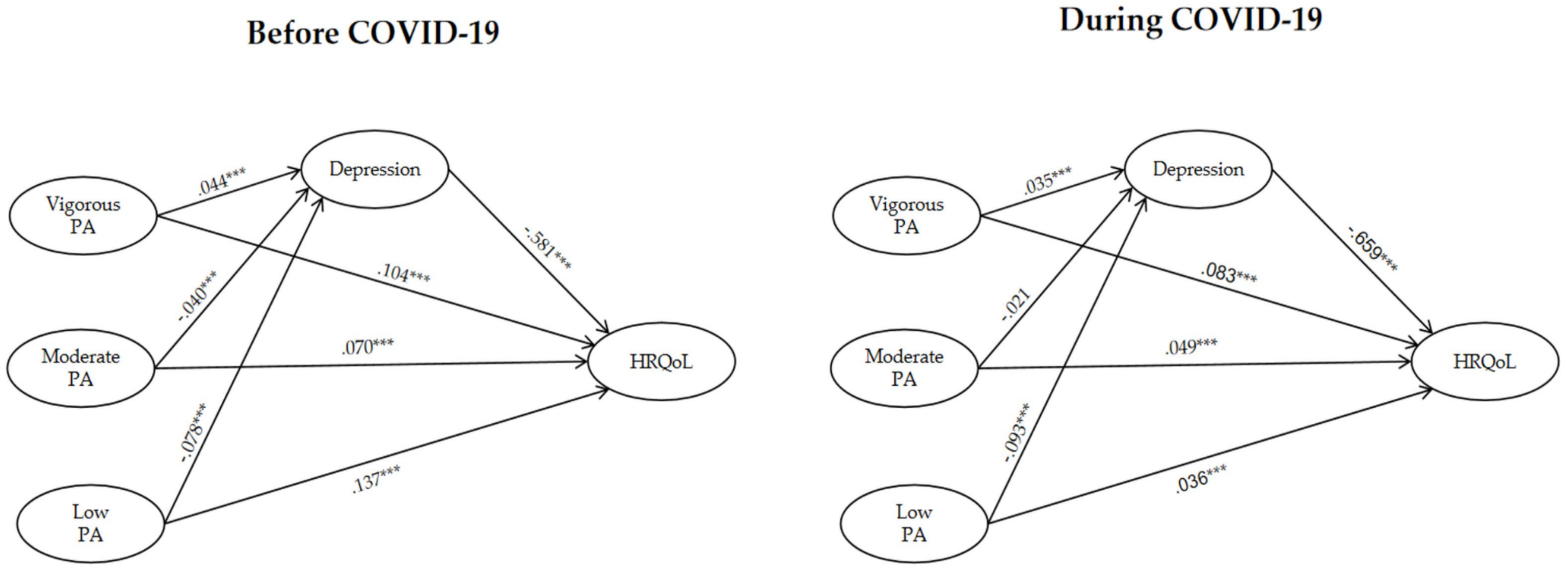
Path analysis for PA, depression, and HRQoL before and during COVID-19.

During COVID-19, low-intensity PA had the strongest relationship to HRQoL (*β* = 0.099). Its indirect impact (*β* = 0.063) was stronger than its direct impact (*β* = 0.036), suggesting that the relationship to HRQoL might primarily operate through depression. Vigorous- and moderate-intensity PA had equally strong impacts (*β* = 0.063), but vigorous-intensity PA exhibited a negative indirect relationship (*β* = −0.020) via stronger depression, whereas moderate-intensity PA had a small positive impact (*β* = 0.014). Overall, the relationships between PA and HRQoL weakened during COVID-19, particularly for low-intensity PA (see Figure 2).

Sobel tests revealed that only low-intensity PA had a significant indirect relationship with HRQoL via depression (Z = 3.25, *p* = 0.001). In contrast, the indirect impacts of vigorous-intensity (Z = −1.692, *p* = 0.090) and moderate-intensity PA (Z = 1.818, *p* = 0.069) were not significant, although the latter approached significance (see Table 4). These results further underscore that low-intensity PA (e.g., walking and yoga) had relatively stronger impacts on mental health and HRQoL. In contrast, vigorous- and moderate-intensity PA might operate on HRQoL through other physiological or psychosocial pathways.

**Table 4:** Sobel test for mediation effects before and during COVID-19.

Path		Z	SE	Sig	Decision
Before COVID-19	Vigorous PA → Depression → HRQoL	−1.692	0.015	0.090	Rejected
	Moderate PA → Depression → HRQoL	1.818	0.013	0.069	Rejected
	Low PA → Depression → HRQoL	3.25	0.014	0.001	Supported
During COVID-19	Vigorous PA → Depression → HRQoL	−1.296	0.017	0.194	Rejected
	Moderate PA → Depression → HRQoL	0.913	0.015	0.361	Rejected
	Low PA → Depression → HRQoL	4.043	0.015	0.000	Supported

## 4 Discussion

The aim of this study was to analyze changes in the health behaviors and health status of older adults in China before and during the COVID-19 pandemic by comparing CHARLS data from 2018 and 2020. The findings indicate that PA of varying intensities relates to chronic disease prevalence, depression, and HRQoL, showing both direct and indirect impacts on health sustainability in the proposed model. From a theoretical perspective, these findings invite an integrated view of SDT, Activity Theory, and Successful Aging Theory, which together help explain the motivational, behavioral, and outcome pathways linking PA to health in later life.

Both before and during the COVID-19 pandemic, older adults who participated in moderate-to-vigorous physical activity (MVPA) had significantly lower prevalence of chronic disease than those who did not. This finding is consistent with existing evidence supporting the role of PA in preventing chronic diseases such as hypertension, diabetes, and dyslipidemia (41, 46, 47). These results further reinforce the importance of sustained MVPA as an important non-pharmacological strategy for chronic disease prevention and management (48, 49). Health sustainability is inherently tied to these findings, as they demonstrate the importance of maintaining regular MVPA throughout a lifespan to reduce the burden of chronic disease.

Interestingly, we found that the prevalence of chronic diseases in participants with low-intensity PA was higher than in non-participants, both before and during the pandemic. Therefore, Hypothesis 1 was partially supported. PA significantly related to chronic disease prevalence, though the direction and strength of this association differed by PA intensity. MVPA showed clear protective associations, while low-intensity PA appeared to reflect compensatory engagement after disease onset rather than a preventive relationship. This finding diverges from the results commonly reported in previous studies and might reflect selection bias and behavioral changes after diagnosis. Specifically, older adults diagnosed with a disease might tend to choose low-intensity activities (such as walking and Tai Chi) to maintain health. From the perspective of SDT, such behavior might reflect externally regulated or health-driven motivation, through which individuals initiate PA in response to illness or medical advice rather than intrinsic interest (50). In this context, low-intensity PA might represent reactive rather than preventive behavior, partly explaining its weaker relationship to chronic disease prevention. This finding aligns with Li (51), who found that low-intensity PA positively related to the prevalence of type 2 diabetes in urban older adults in China who changed their health behaviors according to medical advice after being diagnosed with diabetes. Therefore, our findings provide a new perspective on the management of chronic disease in older adults and offer important insights into the benefits of different levels of PA intensity after a chronic disease diagnosis.

From a theoretical perspective, sustainable health behavior depends not only on PA participation but also on the quality of motivation underlying that behavior. According to SDT, when older adults are able to choose appropriate exercise intensity, experience a sense of competence, and maintain social connections during participation, they are more likely to develop intrinsic motivation and sustain long-term engagement in PA (50). Therefore, public health practitioners should provide more targeted exercise recommendations for older adults with chronic diseases and offer progressive exercise options of varying intensities based on their physical condition. Higher levels of intrinsic motivation might facilitate sustained participation in PA, in turn helping maintain physical function, prevent chronic disease, alleviate depression symptoms, and improve psychological well-being. The result is a positive “motivation-behavior-health” cycle. For example, providing personalized exercise guidance to older adults through community health workers or telemedicine platforms can help them make more appropriate behavioral adjustments after a chronic disease diagnosis, thus avoiding the simple and sometimes counterproductive application of uniform standards.

We also analyzed changes in the prevalence of chronic disease in older adults before and during the COVID-19 pandemic. The results show that the prevalence of each chronic disease increased but that the increase was relatively small. Chronic diseases typically develop gradually over a long period of time due to the combined effects of lifestyle, genetic, and environmental factors (52). The pandemic might have accelerated the progression of the disease or affected disease management to some extent. Primary reasons might include factors such as disruptions in medical services, reduced PA, and increased psychological stress (53, 54). From the perspective of Activity Theory, restrictions during the pandemic might have reduced opportunities for regular physical and social engagement, which are essential for maintaining functional health and daily routines (21). Such disruptions in active engagement might partially explain the observed increases in chronic disease prevalence, even if the short-term changes appear modest.

Beyond the physical health impacts of PA and the pandemic, we observed that depression scores increased significantly during COVID-19 across all groups, consistent with evidence of the adverse mental health impact of major public health crises (55, 56). However, participation in low- and moderate-intensity PA related to lower depression scores than non-participation, supporting evidence that PA can alleviate depression symptoms through neurobiological, psychological, and social mechanisms (57–59). In line with Activity Theory, continued participation in PA can help older adults maintain daily structure, social interaction, and a sense of purpose, all of which are important for emotional stability and psychological well-being. Unexpectedly, older adults engaging in vigorous-intensity PA exhibited higher depression scores than non-participants, diverging from findings that vigorous exercise alleviates depression symptoms (60). This discrepancy might reflect the nature of vigorous-intensity PA in CHARLS: physically demanding occupational or domestic labor, particularly in rural settings, rather than voluntary leisure exercise (42). Such “passive” or necessity-driven PA might induce physical fatigue, emotional burden, and stress rather than mental relaxation, thus limiting potential psychological benefits (61). This notion aligns with evidence of the PA paradox, where occupational PA does not consistently convey the same benefits as leisure-time activity (62). Therefore, Hypothesis 2 was partially supported. Depression symptoms among Chinese older adults differed before and during the pandemic and varied according to PA participation and intensity. Our prediction that PA would relate to less severe depression symptoms held true for low- and moderate-intensity PA, but not for vigorous-intensity PA, where the relationship was reversed. These results highlight the importance of distinguishing between voluntary leisure-time PA and non-leisure or occupational PA in research and policy. Interventions for older adults should prioritize activities that are easy to participate in and of low to moderate-intensity, offering physical and mental benefits, especially under public health restrictions such as those imposed during the COVID-19 pandemic.

We also observed that HRQoL among older adults was generally lower during COVID-19 than before the pandemic, suggesting that the pandemic had a negative impact on overall well-being in older adults. This pattern is well documented in the literature, as lockdowns and social distancing disrupted daily routines and reduced social activities, which are key determinants of HRQoL (63, 64). From the perspective of Activity Theory, such disruptions in routine activities and social participation might weaken role continuity and life engagement, thereby contributing to declines in HRQoL. Furthermore, participants who engaged in PA generally reported higher HRQoL both before and during the pandemic, highlighting the importance of maintaining active engagement in daily life. We also found that although the HRQoL of participants engaging in low-intensity PA declined during the pandemic, low-intensity PA still provided stronger protection than the other levels of intensity, effectively mitigating the negative impact of the pandemic on physical and mental health. Low-intensity PA might improve HRQoL primarily through psychological and behavioral pathways, such as stress reduction, improved sleep quality, and emotional regulation, rather than through direct physiological mechanisms (65, 66). Therefore, Hypothesis 3 was fully supported. HRQoL among Chinese older adults differed before and during the pandemic and varied according to PA participation and intensity. All PA intensities related to higher HRQoL, but low-intensity PA has the strongest impact.

Path analysis revealed that different PA intensities had different impacts on depression levels and HRQoL in older adults. Results show that depression was part of a significant indirect pathway between PA and HRQoL in the low- and moderate-intensity PA groups, while no similar pattern emerged in the vigorous-intensity PA group. Low-intensity PA not only directly related to HRQoL but also indirectly through less severe depression symptoms in older adults, forming a significant “PA-depression-HRQoL” pathway. Further path analysis revealed the relatively stronger role of low-intensity PA, especially during the pandemic, when the indirect impact of low-intensity PA was greater than the direct impact, indicating that under conditions of restricted activity, psychological adjustment might play a greater role than physiological pathways in maintaining HRQoL among older adults. In line with Activity Theory, even low-intensity PA can help older adults maintain daily routines, social interaction, and a sense of engagement, which are critical for emotional stability and life satisfaction, particularly when external activity opportunities are limited (67). This finding underscores the value of low-intensity PA for psychological resilience and HRQoL maintenance under pandemic-related constraints (68). In contrast, the indirect impact of vigorous-intensity PA on HRQoL was negative, suggesting that such activity might offset its potential physiological benefits by exacerbating depression symptoms. Depression symptoms negatively related to HRQoL and might act as an intermediate factor in the relationship between PA and HRQoL in older adults, meaning that changes in mood might counteract the physical benefits of vigorous-intensity activity (16, 69). From the perspective of Successful Aging Theory, these results highlight that psychological well-being is a core component of healthy aging and that improvements in HRQoL depend not only on physical functioning but also on emotional and mental health (70). Therefore, Hypothesis 4 was partially supported, suggesting that depression was involved in an indirect pathway between low to moderate-intensity PA and HRQoL, a role not observed in vigorous-intensity PA. This finding further indicates that low to moderate-intensity PA is crucial for older adults, especially given limited public health resources, and has practical value in promoting sustainable health maintenance.

An integrated theoretical perspective can help explain the mechanism underlying the stronger association between low-intensity PA and HRQoL, particularly during the COVID-19 pandemic. First, from the standpoint of SDT, low-intensity PA is more likely to be autonomously motivated, as it is accessible, less physically demanding, and easier to sustain among older adults. These features are associated with greater intrinsic motivation and better psychological well-being. Second, according to Activity Theory, even low-intensity activity may help maintain daily routines, social engagement, and a sense of purpose, which are critical for emotional stability, especially under conditions of social restriction. Third, from the perspective of Successful Aging Theory, psychological well-being and adaptive capacity are core components of healthy aging, particularly when external stressors limit physical functioning. These mechanisms suggest that while low-intensity PA might have limited physiological effects on chronic disease prevention, it plays a disproportionately important role in promoting mental health and maintaining HRQoL through psychological and behavioral pathways. As the findings show, low-intensity PA showed the strongest overall associations with HRQoL, particularly during the pandemic.

These findings offer new insights into public health policy development, particularly in promoting health sustainability in older adults. From a practical perspective, personalized exercise prescriptions for older adults should prioritize moderate-intensity PA (e.g., brisk walking, Tai Chi, and square dancing), which relates to lower chronic disease prevalence and better overall health. Meanwhile, low-intensity PA remains important for older adults with reduced physical function. Activities that are easy to participate in and have low barriers to entry should be actively promoted, including walking, online-guided square dancing, home workouts using everyday items, and simple indoor aerobic exercises. Combined with basic psychological and social support, these activities might help older adults maintain physical and mental well-being in their home environment. During social restrictions or public health emergencies, low-intensity activities might be a more feasible and appropriate option for vulnerable older adults due to their associations with mental health and HRQoL.

## 5 Conclusion

Chinese older adults experienced a noticeable decline in health during the pandemic, with increased prevalence of hypertension, dyslipidemia, and diabetes, more severe depression symptoms, and a decline in HRQoL. Both before and during the pandemic, MVPA related to lower chronic disease prevalence. Compared to vigorous-intensity PA, moderate-intensity PA showed sustained favorable impacts both before and during the pandemic, relating not only to more favorable chronic disease profiles but also to higher HRQoL and less severe depression symptoms. Notably, low-intensity PA, despite its limited impact on chronic disease prevention, demonstrated the strongest overall relationship to HRQoL through its strong links to mental health. This finding highlights the importance of psychological pathways in shaping health outcomes, particularly under conditions of external constraint such as public health emergencies.

Compared to previous findings based on cross-sectional data or focused on single outcomes, findings from the current study provide longitudinal evidence, based on nationally representative data, of the differential roles of PA intensity and the mediating role of depression in the relationship between PA and HRQoL before and during the pandemic. These findings extend existing literature by demonstrating that the benefits of PA are intensity-specific and context-dependent, with low-intensity PA playing a particularly critical role in promoting health sustainability under constraining conditions.

From a theoretical perspective, the findings suggest the value of integrating SDT, Activity Theory, and Successful Aging Theory into a unified framework, thereby clarifying the motivational, behavioral, and outcome mechanisms through which PA influences health in later life. Importantly, the findings suggest that sustainable health benefits are not solely determined by physiological intensity but depend on the psychological accessibility and adaptability of health behaviors.

From a practical perspective, the findings suggest that public health strategies should move beyond uniform PA recommendations to adopt intensity-specific and context-sensitive approaches. For example, community-based programs can prioritize low- to moderate-intensity activities such as walking groups, Tai Chi, and home-based exercises, particularly for older adults with limited mobility or during periods of social restriction. In addition, digital or remote-guided PA interventions might provide feasible alternatives to maintain engagement under emergency conditions. Creating supportive environments that enhance autonomy, accessibility, and continuity of participation is essential for promoting long-term participation in PA and sustaining health among older adults.

## Data Availability

The original contributions presented in the study are included in the article/supplementary material, further inquiries can be directed to the corresponding author.
